# CF_4_ Capture and Separation of CF_4_–SF_6_ and CF_4_–N_2_ Fluid Mixtures Using Selected Carbon Nanoporous Materials and Metal–Organic Frameworks: A Computational Study

**DOI:** 10.1021/acsomega.1c06167

**Published:** 2022-02-16

**Authors:** Ioannis Skarmoutsos, Emmanuel N. Koukaras, Emmanuel Klontzas

**Affiliations:** † Theoretical and Physical Chemistry Institute, National Hellenic Research Foundation, Vas. Constantinou 48, GR-116 35 Athens, Greece; ‡ Laboratory of Quantum and Computational Chemistry, Department of Chemistry, Aristotle University of Thessaloniki, 54124 Thessaloniki, Greece

## Abstract

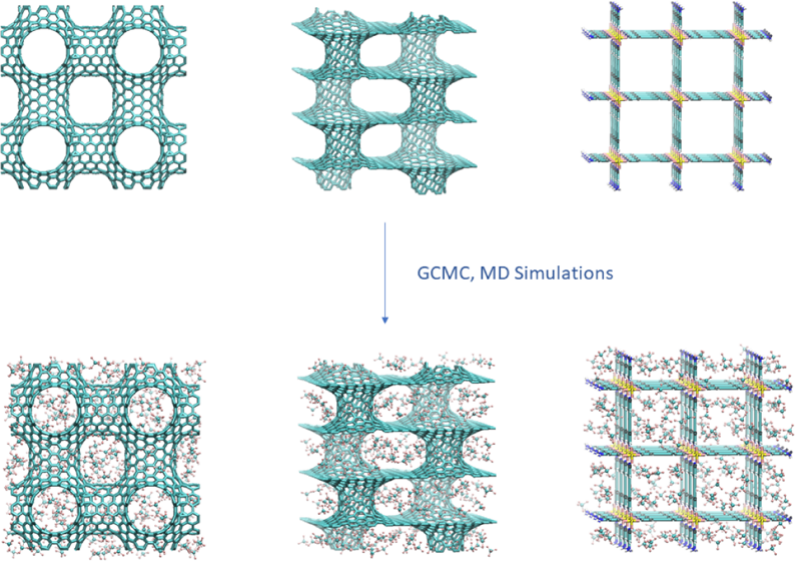

The adsorption of pure fluid carbon tetrafluoride and the separation of CF_4_–SF_6_ and CF_4_–N_2_ fluid mixtures using representative nanoporous materials have been investigated by employing Monte Carlo and molecular dynamics simulation techniques. The selected materials under study were the three-dimensional carbon nanotube networks, pillared graphene using carbon nanotube pillars, and the SIFSIX-2-Cu metal–organic framework. The selection of these materials was based on their previously reported efficiency to separate fluid SF_6_–N_2_ mixtures. The pressure dependence of the thermodynamic and kinetic separation selectivity for the CF_4_–SF_6_ and CF_4_–N_2_ fluid mixtures has therefore been investigated, to provide deeper insights into the molecular scale phenomena taking place in the investigated nanoporous materials. The results obtained have revealed that under near-ambient pressure conditions, the carbon-based nanoporous materials exhibit a higher gravimetric fluid uptake and thermodynamic separation selectivity. The SIFSIX-2-Cu material exhibits a slightly higher kinetic selectivity at ambient and high pressures.

## 1 Introduction

One of the main aims of international environmental treaties and agreements, such as the Kyoto Protocol and the Paris Agreement within the United Nations Framework Convention on Climate Change, is to reduce greenhouse gas emissions in the atmosphere in order to prevent dangerous anthropogenic interference with the climate system.^1,2^ For this reason, six categories of greenhouse gases, sometimes also distinguished as CO_2_^3^ and non-CO_2_ ones,^4^ have been classified among the most potent ones. These greenhouse gases are carbon dioxide (CO_2_), methane (CH_4_), nitrous oxide (N_2_O), hydrofluorocarbons, perfluorocarbons (PFCs), and sulfur hexafluoride (SF_6_).

Among the non-CO_2_ greenhouse gases, tetrafluoromethane (CF_4_), the simplest PFC, has a very long atmospheric lifetime (50,000 years) and a 100 year global warming potential of 6630–7390.^5^ Although its concentration in the atmosphere is low (around 74 ppt), CF_4_ contributes significantly to the greenhouse effect.^6^ Therefore, its capture or recovery from CF_4_/N_2_ mixtures from industrial emission sources,^7,8^ mainly associated with the aluminum production and semiconductor fabrication processes, is considered important for the reduction of global warming. Moreover, the recycle of CF_4_ from CF_4_/N_2_ mixtures is also important in a wide range of applications involving a positron trap^9^ since these mixtures are used as buffer gases for the cooling of positrons.^10^

The recovery and recycle of CF_4_ are also highly important in mixtures with SF_6_ having significant technological applications. Mixtures of CF_4_ with SF_6_ are used as insulating and arc-quenching media^11−18^ at very low temperatures, down to −50 °C, especially in circuit breakers operating at these low temperatures.^11^ In these mixtures, CF_4_ is added in order to avoid liquefaction of SF_6_ gas at low temperatures. These mixed gas circuit breakers are installed in converting stations, where alternating current is converted to direct current. The direct current is subsequently transmitted over long distances on a high-voltage direct current transmission line. According to the recent climate negotiation in Paris, a speedy implementation of these networks is a main path to introduce renewable energy and to harmonize power markets.

To achieve an efficient CF_4_ capture, several technologies based on thermal decomposition, plasma treatment, absorption, adsorption, cryogenic recovery, and membrane separation have been developed. Among them, physical adsorption using efficient nanoporous adsorbents^6,7,19−33^ is considered as the most competitive technology for capturing CF_4_ due to its low energy consumption, low cost, and easy management. However, only some limited studies in the literature are devoted to CF_4_ capture using nanoporous adsorbents. In this respect, we decided to further explore the CF_4_ capture and the separation of SF_6_–CF_4_ and CF_4_–N_2_ fluid mixtures using some selected carbon-based nanoporous materials and metal–organic frameworks (MOFs), which have been found in our previous studies^34−36^ to be very efficient for SF_6_ capture and SF_6_–N_2_ fluid mixture separation. These previous studies^34,36^ had revealed that three-dimensional interconnected single-wall carbon nanotube networks and pillared graphene nanostructures, consisting of parallel graphene layers stabilized by carbon nanotubes placed vertically to the graphene planes, exhibit a significant SF_6_ uptake and high adsorption selectivity for SF_6_ over N_2_, in comparison with the best performing materials in the literature. Our studies^35^ also revealed that the SIFSIX-2-Cu MOF exhibits high thermodynamic and kinetic adsorption selectivity for SF_6_ over N_2_.

Based on these findings, the aim of the present study was to further explore the efficiency of these different types of nanoporous materials in capturing CF_4_ and separating SF_6_–CF_4_ and CF_4_–N_2_ fluid mixtures, using a combination of force field-based grand canonical Monte Carlo (GCMC) and molecular dynamics (MD) simulations. This paper is organized as follows: the employed computational methodology is presented in Section 2, the results and corresponding discussion in Section 3, and finally, the concluding remarks are summarized in Section 4.

## 2 Computational Methods

As mentioned in the introduction, two types of carbon-based nanoporous materials were investigated in the framework of the present study. The first type is a porous nanotube network (PNN),^37^ composed of (8,8) single-walled carbon nanotubes which are connected through junctions, forming a three-dimensional cubic carbon nanotube network with an intertube distance of 11 Å and a corresponding three-dimensional porous network. The second type is the PILS pillared graphene that^34,38^ consisted of parallel graphene layers stabilized by carbon nanotubes placed vertically to the graphene planes. The pillared graphene nanostructure investigated in the present study is composed of (6,6) single-walled carbon nanotubes, with a nanotube diameter of 8.142 Å, which act as pillars between adjacent graphene layers. In this specific nanostructure, the interlayer distance is 11.2 Å and the intertube one is 20.9 Å. The supercells used in our simulations comprised 1 × 1 × 1 unit cells in the case of PNN and 1 × 1 × 2 unit cells in the case of PILS. The PNN supercell is cubic with a 41 Å dimension, whereas the PILS supercell is orthorhombic, with dimensions 42.60 Å × 39.35 Å × 44.84 Å.

The third nanoporous material taken into account in the present investigation is the [Cu(4,4′-dipyridylacetylene)_2_(SiF_6_)]_*n*_ MOF, named SIFSIX-2-Cu, resembling pillared square grids with a pore dimension of 13.05 Å. This particular MOF was synthesized by pillaring two-dimensional nets of organic ligands and metal nodes with hexafluorosilicate (SiF_6_^2–^) anions to form three-dimensional networks with primitive cubic topology.^39^ The SIFSIX-2-Cu supercell was constituted by a 3 × 3 × 5 replica of its unit cell, which was geometry optimized in our previous studies at the periodic density-functional theory level.^35^ As mentioned in our previous studies, the SIFSIX-2-Cu supercell was saturated by adding terminal −NH_2_ groups, and the charges of the terminal atoms were adjusted in order to give a total zero charge to the supercell.^35^ The supercell dimensions are 41.25 Å × 41.25 Å × 42.01 Å. Further details about the special characteristics of all the investigated nanomaterials can be found in our previous studies.^34−36^

A combination of force field-based GCMC and MD simulations was employed to explore the adsorption of pure fluid CF_4_ in PNN, PILS, and SIFSIX-2-Cu and the separation of the CF_4_/SF_6_ (two bulk molar compositions CF_4_/SF_6_: 1:1 and 9:1) and CF_4_/N_2_ binary mixtures (bulk molar composition CF_4_/N_2_: 1:9). These simulations were performed at 303 K and in the pressure range 0.1–20 bar. The well-established all-atom rigid potential model for SF_6_ developed by Dellis and Samios^40^ and the TraPPE potential model for N_2_^41^ have been selected to be employed in our simulation studies. According to the literature,^40,41^ these models provide very accurate description of fluid properties of SF_6_ and N_2_ under a wide range of thermodynamic conditions and the vapor–liquid coexistence curve and critical points of the pure systems. The intramolecular geometries, charges, and Lennard-Jones 12–6 potential parameters of all the interaction sites in the potential model of SF_6_ can also be found in a previous study of one of the authors in the literature,^35^ whereas the corresponding ones for N_2_ can be found in the previous study of Potoff and Siepmann.^41^ The OPLS rigid potential model^42^ has been employed for CF_4_. The intramolecular geometries, charges, and Lennard-Jones 12–6 potential parameters of all the interaction sites in the employed potential models of CF_4_, SF_6_, and N_2_ are presented in Table 1. The carbon atoms in the PNN and PILS materials are not charged and interact via a Lennard-Jones 12–6 potential having ε_CC_ = 28.2 K and σ_CC_ = 3.4 Å. The partial charges and Lennard-Jones parameters corresponding to each type of atom in the SIFSIX-2-Cu material are also presented in our previous studies.^35^

**Table 1:** Partial Charges and Lennard-Jones 12–6 Parameters Corresponding to Each Type of Interaction Site in the Employed Potential Models of CF_4_, SF_6_, and N_2_^a^

interaction site	*q* (|e|)	σ (Å)	ε (K)
CF_4_			
C	0.48	3.50	48.8126
F	–0.12	2.95	26.6708
SF_6_			
S	0.66	3.228	165.14
F	–0.11	2.947	27.02
N_2_			
N	–0.482	3.31	36.0
COM	0.964		

a C–F bond length: 1.332 Å, S–F bond length: 1.564 Å, and N–N bond length: 1.1 Å.

The reproducibility of the vapor–liquid equilibrium (VLE) curve is of foremost importance during adsorption studies. Thus, in order to further validate the selected force field for CF_4_ and SF_6_, the VLE *T*–ρ curves have been calculated using *NVT* Gibbs ensemble Monte Carlo (GEMC) simulations^43^ and compared to available experimental data.

The RASPA Monte Carlo simulation code^44^ was used in order to perform the GCMC and GEMC simulations. The fugacities of the adsorbed species, which were used as inputs for the GCMC simulations, were calculated by employing the Peng–Robinson equation of state.^45^ For each simulated thermodynamic condition, 10^5^ Monte Carlo cycles were performed for the equilibration and production runs. In these Monte Carlo cycles, different types of trial moves were attempted, including creation or deletion of molecules, translation or rotation, and exchange of the molecular identity. Note that the calculated error bars in the calculated fluid uptakes in the case of the GCMC simulations were in the range of about 1%. The corresponding error bars in the calculated gas and liquid densities in the cases of the GEMC simulations were in the range 0.4–2.0%, with the largest values observed under thermodynamic conditions approaching the critical point. *NVT*-MD simulations were subsequently carried out to calculate the self-diffusion coefficients of the components of the investigated adsorbed mixtures at the ambient pressure of 1 bar and the high pressure of 20 bar. The MD simulations were performed for all the adsorbed mixtures corresponding to each one of the investigated nanoporous materials. The integration of the equations of motion was achieved by employing the well-established leapfrog-type Verlet algorithm^46^ and using an integration time step of 1 fs. The intramolecular geometry of SF_6_, CF_4_, and N_2_ molecules was constrained using the quaternion formalism.^46^ A Nose–Hoover thermostat with a temperature relaxation time of 0.5 ps was used to constrain the temperature during the simulations.^47^ A 12 Å cutoff was used to treat the short-range interactions, and long-range corrections for the Lennard-Jones interactions were also taken into account in all MD and MC simulations. Test simulation runs, employing higher cutoffs (15 and 18 Å), have shown that when using the long-range corrections for the Lennard-Jones interactions, the results obtained converge for all the investigated cutoffs. The long-range electrostatic interactions were also treated using the standard Ewald summation method.^46^ All MD simulations were run for 5 ns, after a 1 ns equilibration period, using the DL_POLY simulation code.^48^ During the simulations, the intramolecular geometries of the adsorbents were kept rigid.

## 3 Results and Discussion

### 3.1 VLE of CF_4_ and SF_6_

**Figure 1:**
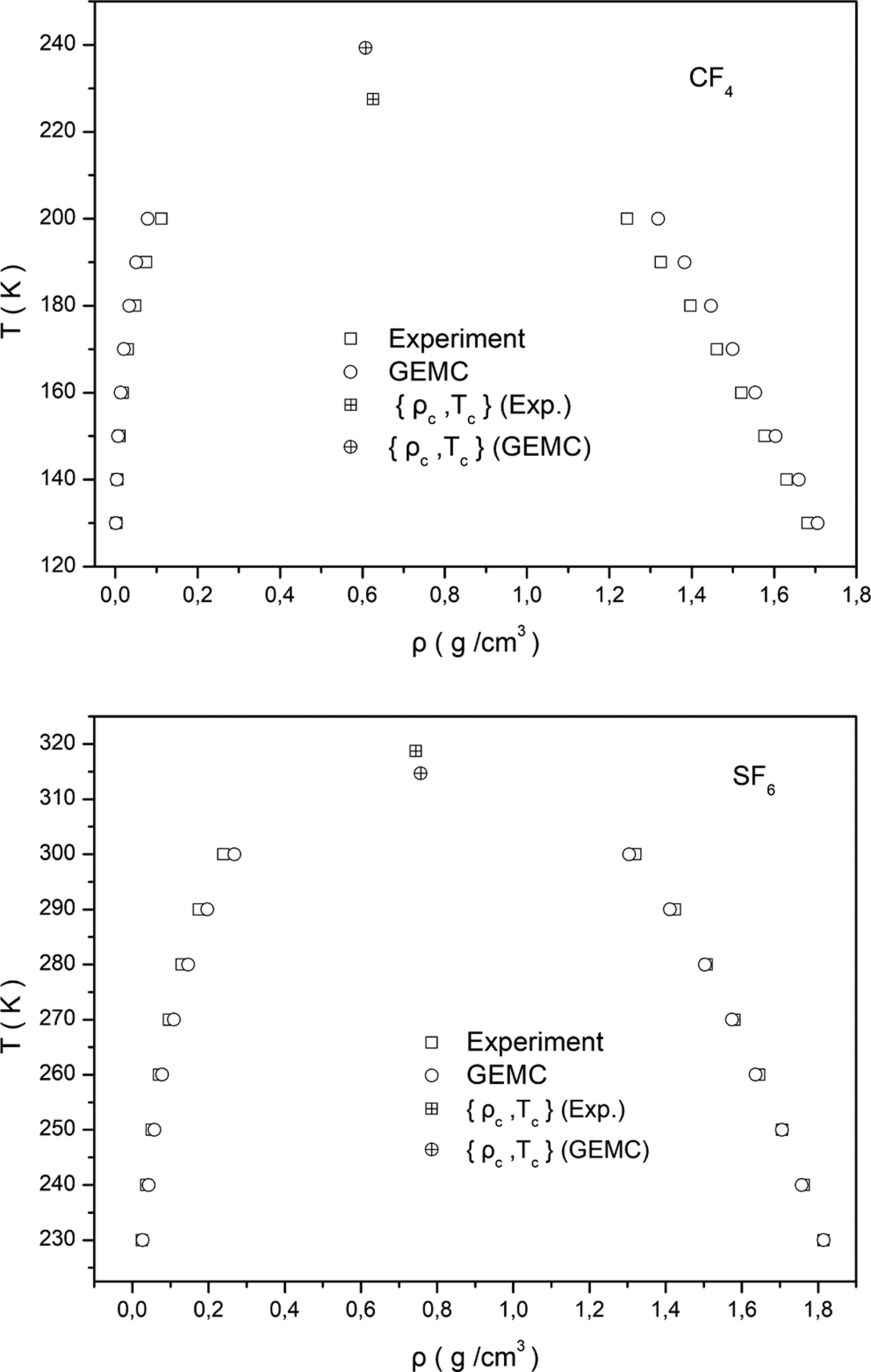
Calculated, from GEMC simulations, *T*–ρ vapor–liquid coexistence curves of pure CF_4_ and SF_6_, plotted together with the experimental ones. The estimated critical points are also presented in comparison with the experiments.

The *NVT*-GEMC simulations for the calculation of the VLE *T*–ρ curves were performed at eight experimentally available coexistence points in the temperature range 130–200 K for pure CF_4_ and in the range 230–300 K for pure SF_6_. The simulated systems consisted of 320 CF_4_ and SF_6_ molecules, equally distributed to the two simulated systems. The molecules in the initial configurations in each subsystem were randomly placed. The density of the two subsystems was set to (ρ_l_ + ρ_v_)/2, where ρ_l_ and ρ_v_ are the experimental values of the density of the liquid and vapor phase at each investigated temperature, respectively. The calculated *T*–ρ vapor–liquid coexistence curves for pure CF_4_ and SF_6_ are presented in Figure 1, along with the experimental ones.

Apparently, the good agreement between the calculated and experimental data can be clearly observed. Moreover, the critical density and temperature of CF_4_ and SF_6_ were estimated by using the well-known critical scaling relation and the law of rectilinear diameters^49^
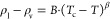
1
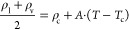
2

The estimated critical values (*T*_c_, ρ_c_) for CF_4_ were *T*_c_ = 239.3 K and ρ_c_ = 0.6078 g/cm^3^, which are in reasonable agreement with the experimental ones (227.5 K, 0.6257 g/cm^3^) and much closer to the experiment compared to the values predicted by other force fields of CF_4_, presented in previous studies in the literature.^50^ Similarly, the estimated critical values (*T*_c_, ρ_c_) for SF_6_ were *T*_c_ = 314.7 K and ρ_c_ = 0.757 g/cm^3^, which are also in good agreement with the experimental ones (318.7 K, 0.743 g/cm^3^).^40^ Note also that the critical exponent β in eq 1 has been estimated to be 0.3105 and 0.3119 in the cases of CF_4_ and SF_6_, respectively. Consequently, the employed force fields of CF_4_ and SF_6_ provide realistic descriptions of the VLE, which according to the literature,^51^ is of paramount importance in adsorption studies.

### 3.2 Pure CF_4_ Adsorption

**Figure 2:**
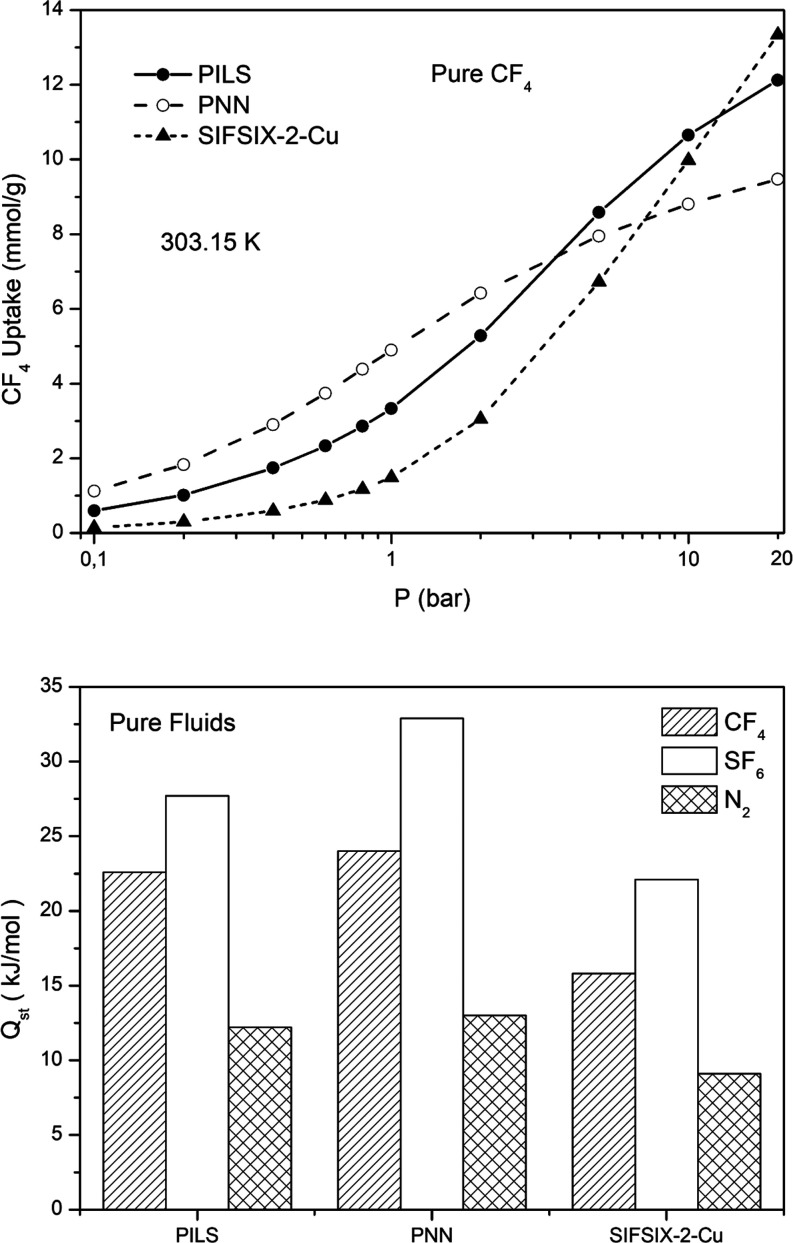
Gravimetric adsorption isotherms of pure CF_4_ at 303.15 K up to 20 bar (top) and isosteric heat of adsorption in the low coverage for CF_4_, SF_6_, and N_2_ (bottom).

**Figure 3:**
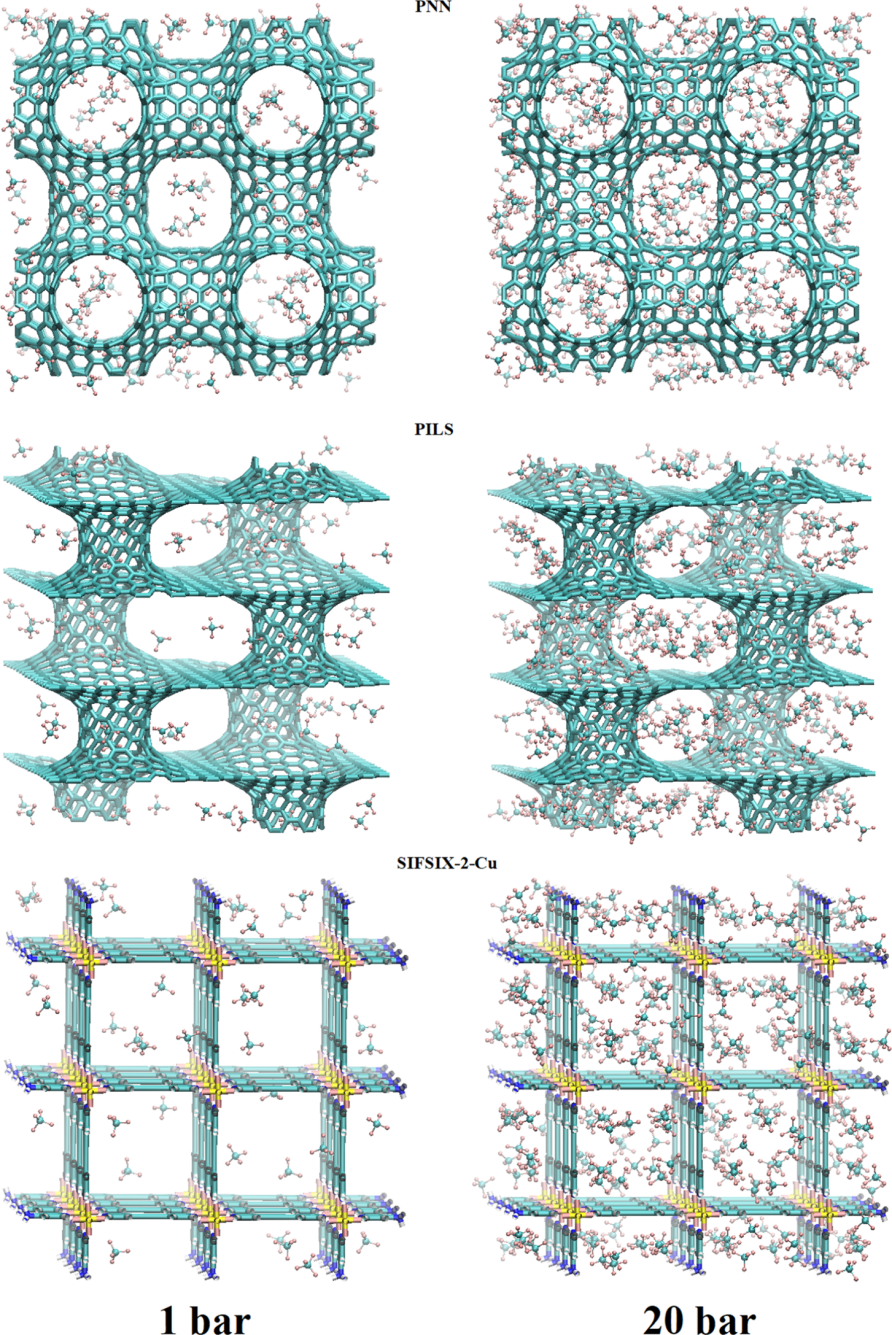
Representative snapshots, depicting the adsorbed CF_4_ molecules in the investigated PNN, PILS, and SIFSIX-2-Cu materials at 303.15 K and *P* = 1, 20 bar.

The gravimetric adsorption isotherms of pure CF_4_ at 303.15 K and in the pressure range 0.1–20 bar for all the investigated materials are illustrated in Figure 2. Apparently, the CF_4_ gravimetric uptake is higher in the case of PNN at the low-pressure range, up to about 5 bar. However, at higher pressures, the gravimetric uptake in the case of PILS and SIFSIX-2-Cu is higher due to the larger free volume of these materials. For instance, at ambient pressure (1 bar), the calculated CF_4_ uptake in the PNN, PILS, and SIFSIX-2-Cu materials is 4.89, 3.33, and 1.49 mmol/g, respectively. These values are among the highest reported regarding the uptake of CF_4_ at ambient pressure, when compared to previous studies for several nanoporous materials in the literature.^32^ At 20 bar, the corresponding values for PNN, PILS, and SIFSIX-2-Cu are 9.48, 12.12, and 13.34 mmol/g, respectively. Representative snapshots, depicting the adsorbed CF_4_ molecules in the investigated materials at 1 and 20 bar, are presented in Figure 3. Within the pressure range studied, it can be deduced that the saturation has not been achieved for any of the adsorbents considered here. To evaluate the interaction strength between the adsorbents and the adsorbates, the isosteric heat of adsorption (*Q*_st_) was calculated at low coverage, corresponding to the pressure of 0.1 bar, which is also presented in Figure 2. The calculated isosteric heat of adsorption of pure CF_4_, obtained in the framework of this study, is presented together with the calculated values for pure SF_6_ and N_2_, which were obtained in our previous studies.^35,36^ Regarding pure CF_4_ adsorption, the highest predicted isosteric heat of adsorption at low coverage is observed in the case of PNN (24.0 kJ/mol), followed by PILS (22.6 kJ/mol), whereas the lowest value of *Q*_st_ corresponds to SIFSIX-2-Cu (15.8 kJ/mol). The higher uptake of CF_4_ at low pressures in the case of PNN can be explained in terms of the calculated *Q*_st_ of pure CF_4_ at low coverage. The trends observed for the values of *Q*_st_ corresponding to each one of the investigated materials are consistent with the corresponding trends regarding the CF_4_ adsorption at low pressures, further confirming that the CF_4_ adsorption at low pressures is thermodynamically driven. The same trends have also been observed in the case of the pure SF_6_ and N_2_ adsorption. Nevertheless, in the case of SF_6_, the calculated *Q*_st_ values are higher in comparison with CF_4_. The corresponding *Q*_st_ values for PNN, PILS, and SIFSIX-2-Cu in the case of pure SF_6_ adsorption are 32.9, 27.7, and 22.1 kJ/mol, respectively. In the case of pure N_2_ adsorption, the corresponding *Q*_st_ values are significantly lower. The calculated *Q*_st_ values for PNN, PILS, and SIFSIX-2-Cu in the latter case are 13.0, 12.2, and 9.1 kJ/mol, respectively.

### 3.3 Separation of CF_4_/SF_6_ Fluid Mixtures

**Figure 4:**
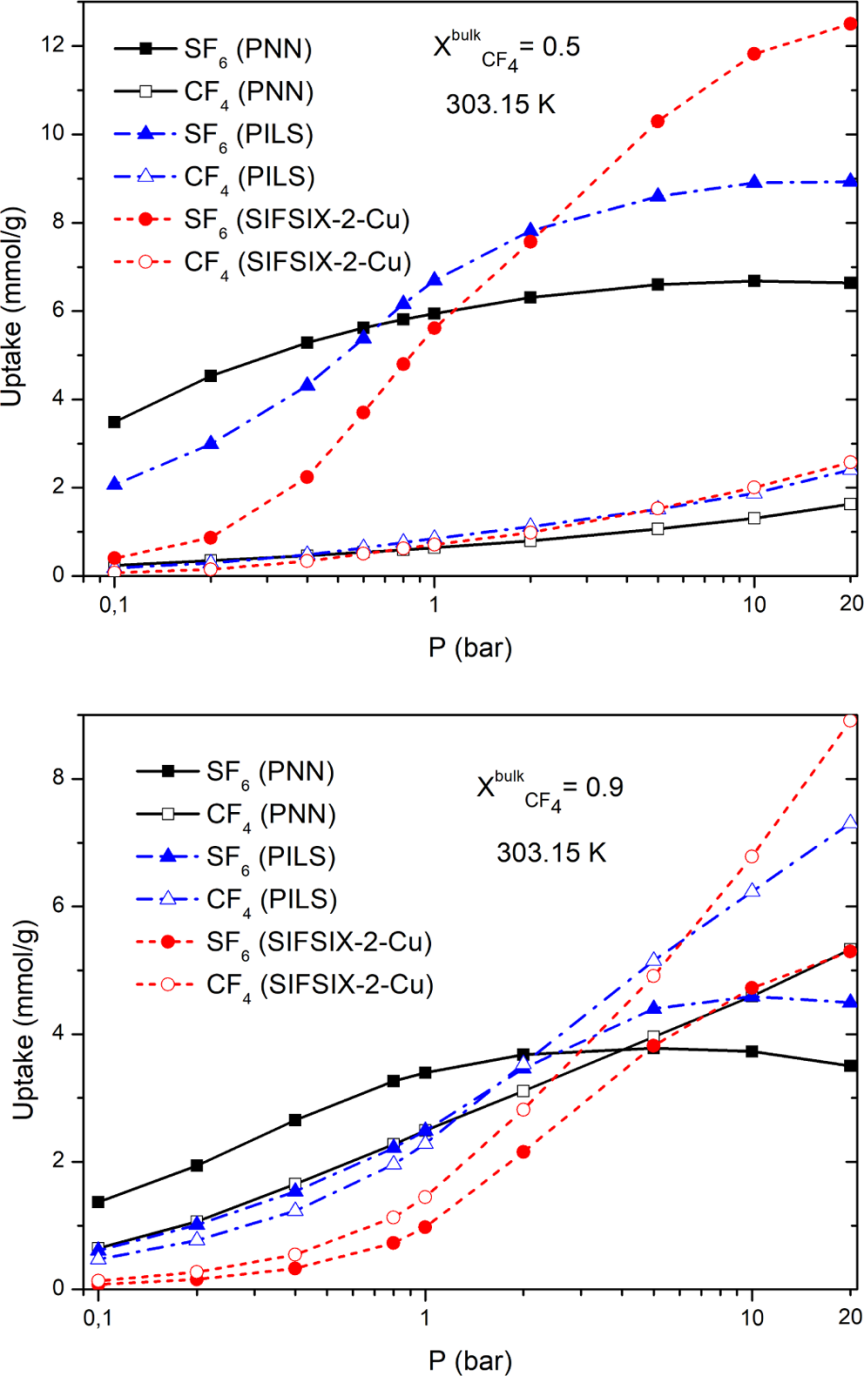
Calculated co-adsorption isotherms of CF_4_/SF_6_ fluid mixtures ().

Subsequently, as mentioned in Section 2, GCMC simulations were performed in order to calculate the co-adsorption isotherms of CF_4_/SF_6_ fluid mixtures. Two bulk molar compositions (CF_4_/SF_6_: 1:1 and 9:1) were taken into account in our calculations, and the results obtained for the PNN, PILS, and SIFSIX-2-Cu materials are presented in Figure 4.

Apparently, the molar fraction of the binary mixture influences the co-adsorption behavior of the adsorbents. In the case of the equimolar mixture, the SF_6_ co-adsorption is much more favored at low pressures, especially in the cases of PNN and PILS. This finding is consistent with the fact that the isosteric heat of adsorption of pure SF_6_ at low coverage is higher in comparison with CF_4_. As the pressure increases, we observe an increase in the loading of CF_4_ that keeps the loading of SF_6_ almost constant for PNN and PILS. In contrast, the sulfur hexachloride loading of SIFSIX-2-Cu exhibits a continuous increase and surpasses the corresponding loading of the carbon-based adsorbents. This reveals that the thermodynamically driven favored adsorption of SF_6_ becomes less pronounced in higher pressures, as attributed to two reasons. The first one is related to the larger size and kinetic diameter of SF_6_ with respect to CF_4_. Additionally, size- and shape-dependent packing effects start to play an important role. The same reasons have a dominant role in the case of the binary mixture containing 10% sulfur hexafluoride. For this mixture composition, increased loading for SF_6_ is observed in the pressure range below 2 bar, which is more pronounced for PNN and PILS. At higher pressures, there is a clear enhancement of the CF_4_ loading, exceeding the loading of SF_6_ for the adsorbents under study. This in an indication of the high competitiveness of both molecules to occupy the available free volume, leading to the displacement of SF_6_ at high pressures. Thus, the selectivity will be reduced at high pressures.

**Figure 5:**
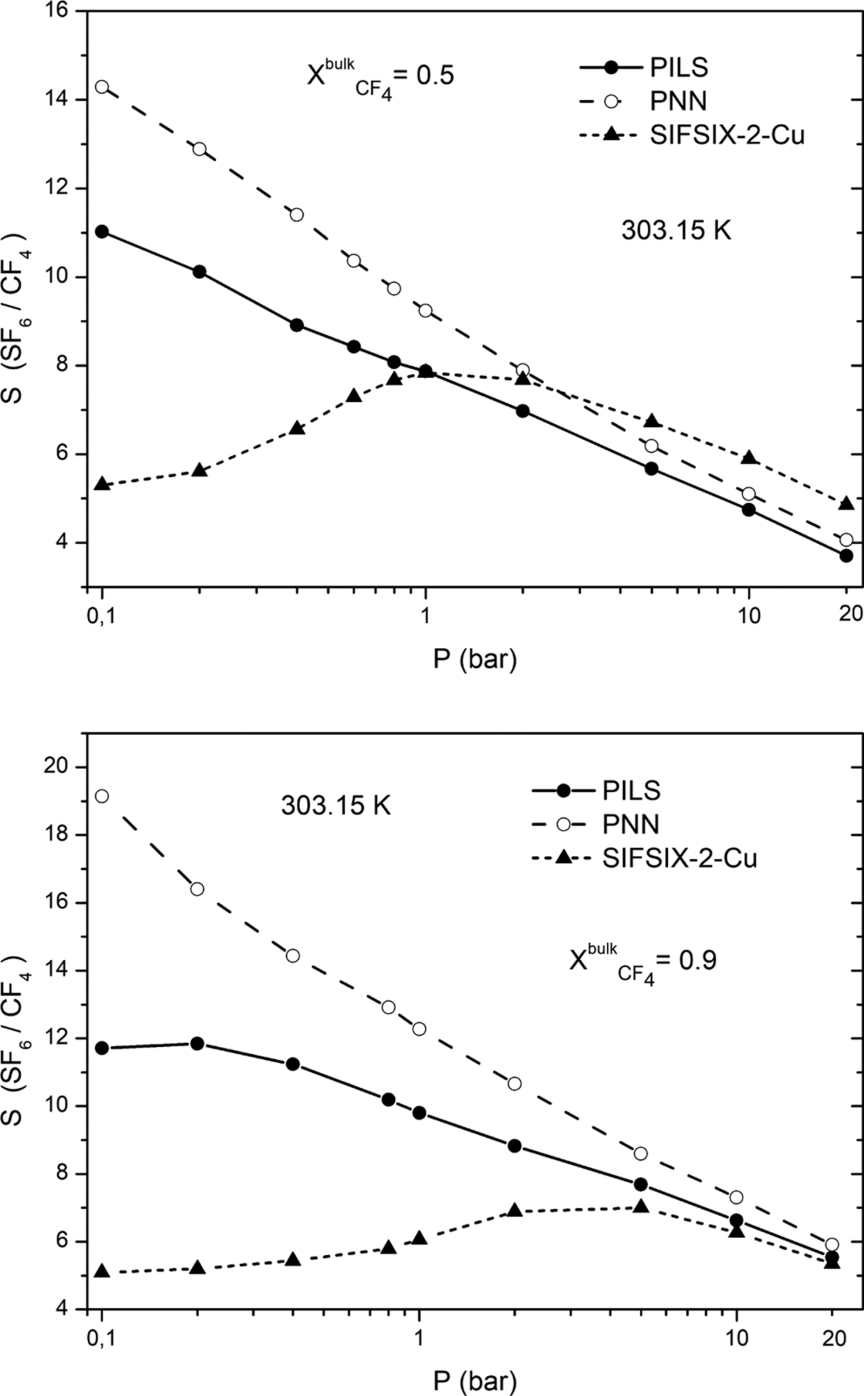
Pressure dependence of the calculated thermodynamic adsorption selectivity  for the investigated fluid mixture compositions at 303.15 K.

The trends observed in the calculated co-adsorption isotherms coupled with the values obtained for *Q*_st_ are more clearly reflected on the calculated values of the thermodynamic adsorption selectivity 
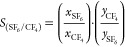
3where *x*_i_ and *y*_i_ are the molar fractions of each component i (i = SF_6_, CF_4_) in the adsorbed and bulk phases, respectively. The calculated thermodynamic adsorption selectivity  for the investigated fluid mixture as a function of pressure at 303.15 K is presented in Figure 5. The thermodynamic adsorption selectivity decreases with the increase in the pressure in the cases of PNN and PILS for both mole fractions of CF_4_ (0.5 and 0.9). The selectivity values observed in both cases are quite similar, with the ones corresponding to PNN being slightly higher. Interestingly, in the case of SIFSIX-2-Cu, the selectivity reaches a maximum value, which is observed at 2 and 5 bar in the cases where the bulk mole fraction of CF_4_ is 0.5 and 0.9, respectively. A similar pressure dependence of the selectivity for the SF_6_/N_2_ separation was also observed for the SIFSIX-2-Cu nanoporous material^35^ and the FAU-ZTC zeolite,^52^ which has a similar pore diameter with SIFSIX-2-Cu. The appearance of such a maximum, which has been observed for both the investigated bulk mixture compositions, can be interpreted in terms of competitive adsorption phenomena at high pressures. At these pressures, the adsorption of CF_4_ in the nanopores is facilitated due to its smaller kinetic diameter in comparison with SF_6_. On the other hand, at lower pressures, the separation mechanism is mainly a thermodynamically driven one.^35,52^ The shift of the selectivity maximum at higher pressures observed for the CF_4_/SF_6_ mixture with a 9:1 bulk molar composition can also be attributed to the lower bulk composition of SF_6_. This lower bulk composition of SF_6_ leads to a more pronounced increase in the slope of the uptake of CF_4_ with pressure at higher pressures in comparison with SF_6_, as it can also be observed in Figure 4, resulting in a lower  selectivity due to packing effects.

MD simulations were further performed in the canonical *NVT* ensemble, using the calculated SF_6_ and CF_4_ uptakes corresponding to 1 and 20 bar, respectively, and the bulk mixture composition with , to explore the diffusivity of the guest molecules in the PNN, PILS, and SIFSIX-2-Cu materials. The self-diffusion coefficients of SF_6_ and CF_4_ were calculated using the well-known Einstein relation applied to the mean-square displacements for both guests averaged over all the MD trajectories and using a multi-time step origin. The calculated self-diffusion coefficients are presented in Table 2.

**Table 2:** Calculated Self-Diffusion Coefficients of the Adsorbed SF_6_ and CF_4_ Molecules in the Investigated Nanomaterials, Corresponding to the Bulk Fluid Mixture with  and Pressures of 1 and 20 bar, Respectively

material	*D*(SF_6_) (10^–9^ m^2^/s)	*D*(CF_4_) (10^–9^ m^2^/s)	*D*(CF_4_)/*D*(SF_6_)
*P* = 1 bar,			
PILS	7.55	12.44	1.65
PNN	2.46	2.91	1.18
SIFSIX-2-Cu	2.30	6.81	2.96
*P* = 20 bar,			
PILS	1.91	2.85	1.49
PNN	0.98	1.37	1.52
SIFSIX-2-Cu	2.12	3.85	1.82

From this table, we can see that the relative percentage decrease in the self-diffusion of the adsorbed SF_6_ and CF_4_ molecules upon the increase in the pressure is more pronounced in the case of the PNN and PILS materials. However, the ratio *D*(CF_4_)/*D*(SF_6_) is higher in the case of SIFSIX-2-Cu, signifying that the kinetic separation of the adsorbed mixture components is slightly more favored in SIFSIX-2-Cu.

### 3.4 Separation of CF_4_/N_2_ Fluid Mixtures

**Figure 6:**
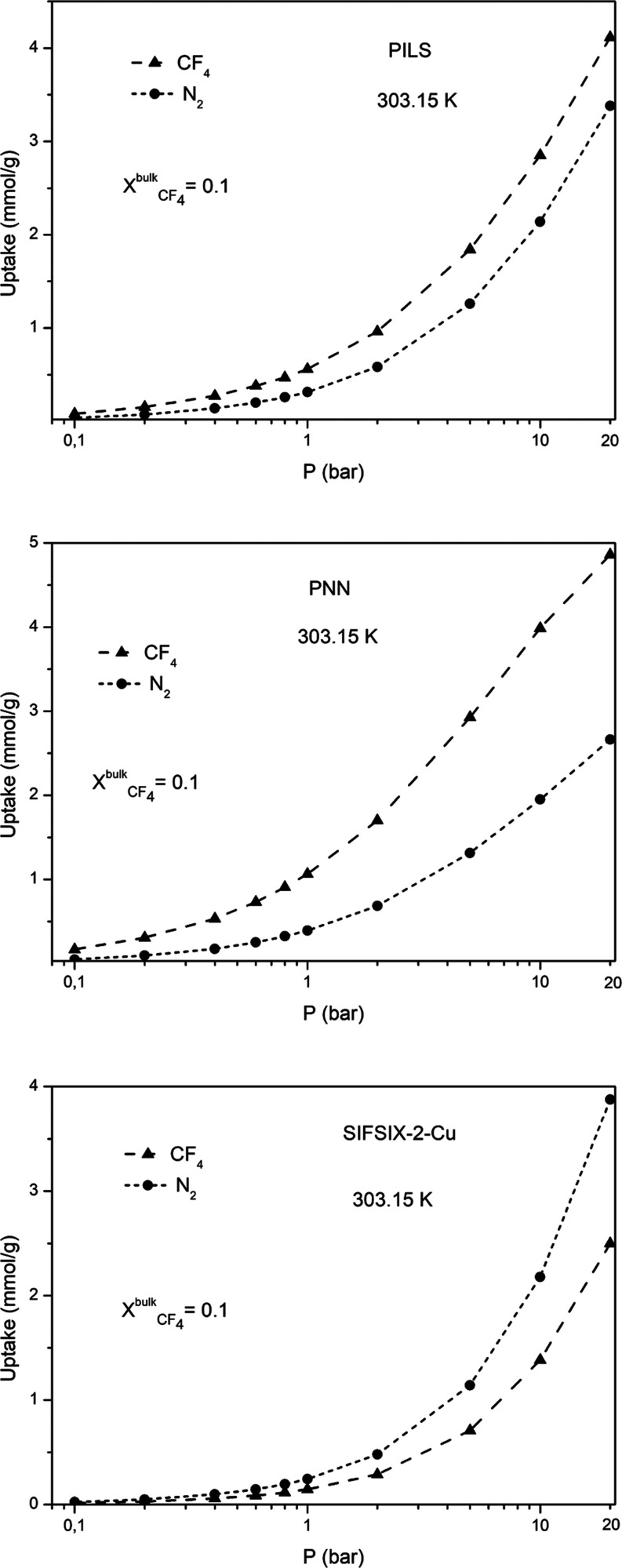
Calculated co-adsorption isotherms of CF_4_/N_2_ fluid mixtures ().

The co-adsorption isotherms of a CF_4_/N_2_ fluid mixture (with bulk molar composition CF_4_/N_2_: 1:9) were also investigated in the present treatment, and the results obtained for the PNN, PILS, and SIFSIX-2-Cu materials are presented in Figure 6. From this figure, we can see that the actual gravimetric uptakes of each one of the mixture components are quite low at ambient pressures, and only at the high-pressure region, they exhibit values in the range 2–5 mmol/g.

**Figure 7:**
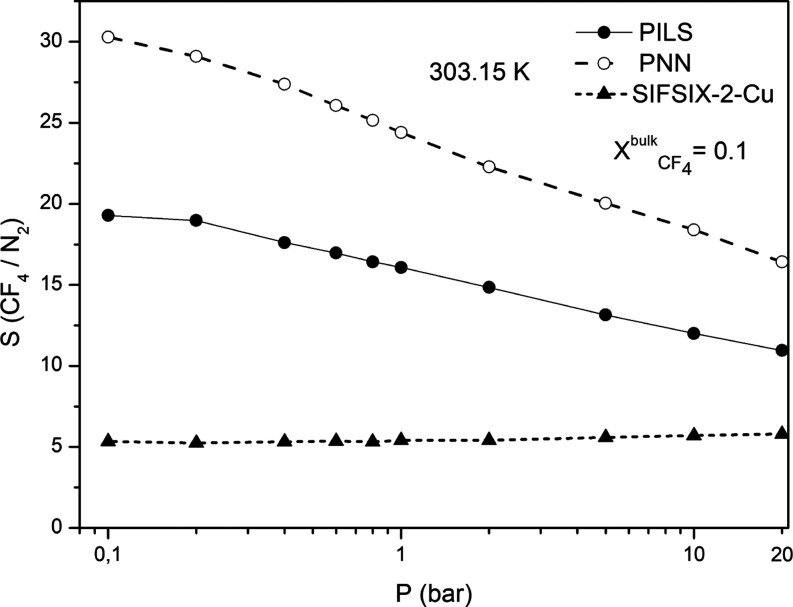
Pressure dependence of the calculated thermodynamic adsorption selectivity  for the investigated fluid mixture composition at 303.15 K.

The calculated values of the thermodynamic adsorption selectivity  are also presented in Figure 7. From these figures, we can see that the thermodynamic selectivity  exhibits the highest values in the case of the carbon-based nanoporous materials, particularly for the PNN. This finding is also consistent with our previous studies,^34^ which had revealed that the thermodynamic separation selectivity of SF_6_/N_2_ mixtures is significantly enhanced in the case of the PNN and PILS materials. However, the selectivity values observed in the case of the CF_4_/N_2_ mixture are significantly lower in comparison with the ones obtained for the SF_6_/N_2_ mixture with the same molar composition. This finding is also consistent with the lower isosteric heat of adsorption values *Q*_st_ for CF_4_ in comparison with SF_6_. From Figure 7, it can also be observed that for both the PNN and PILS materials, the selectivity  values decrease with the increase in the pressure. On the other hand, in the case of SIFSIX-2-Cu, the selectivity remains almost constant along the investigated pressure range, exhibiting a value around five. The much lower selectivity value in the case of SIFSIX-2-Cu can also be attributed to the fact that the actual uptake value of CF_4_ along the investigated pressure range is lower in comparison with the one corresponding to N_2_, whereas the opposite behavior is observed in the case of PNN and PILS. All these findings clearly indicate that the thermodynamic adsorption selectivity in the case of the CF_4_/N_2_ fluid mixture is more pronounced for the carbon-based nanoporous materials, particularly in the case of PNN.

Due to the very low uptake of the mixture components at 1 bar, MD simulations were performed in the canonical *NVT* ensemble using the calculated CF_4_ and N_2_ uptakes corresponding to 20 bar and the bulk mixture composition with , to explore the diffusivity of the guest molecules in the PNN, PILS, and SIFSIX-2-Cu materials. The calculated self-diffusion coefficients of CF_4_ and N_2_ are presented in Table 3.

**Table 3:** Calculated Self-Diffusion Coefficients of the Adsorbed N_2_ and CF_4_ Molecules in the Investigated Nanomaterials, Corresponding to the Bulk Fluid Mixture with  and Pressures of 20 bar

*P* = 20 bar,			
material	*D*(N_2_) (10^–9^ m^2^/s)	*D*(CF_4_) (10^–9^ m^2^/s)	*D*(N_2_)/*D*(CF_4_)
PILS	25.92	13.89	1.87
PNN	9.19	5.58	1.65
SIFSIX-2-Cu	27.65	7.48	3.70

From this table, it can be clearly observed that the ratio *D*(N_2_)/*D*(CF_4_) is higher in the case of SIFSIX-2-Cu, indicating that the kinetic separation of the adsorbed mixture components is more favored in SIFSIX-2-Cu, as in the case of the CF_4_–SF_6_ mixture.

## 4 Conclusions

The adsorption of pure fluid carbon tetrafluoride and the separation of CF_4_–SF_6_ and CF_4_–N_2_ fluid mixtures, using three-dimensional carbon nanotube networks (PNN), pillared graphene with carbon nanotube pillars (PILS), and the SIFSIX-2-Cu MOF, were investigated by employing a combination of Monte Carlo and MD simulation techniques. These particular nanoporous materials were selected based upon their very satisfactory performance for SF_6_ capture and SF_6_–N_2_ fluid mixture separation, which was revealed in our previous studies.

The results obtained regarding pure CF_4_ adsorption have revealed that the highest predicted isosteric heat of adsorption at low coverage is observed in the case of PNN (24.0 kJ/mol), followed by PILS (22.6 kJ/mol), whereas the lowest value of *Q*_st_ corresponds to SIFSIX-2-Cu (15.8 kJ/mol). These trends are consistent with the corresponding trends regarding the gravimetric uptake of pure CF_4_ adsorption at low pressures, further confirming that the CF_4_ adsorption at low pressures is thermodynamically driven. However, at higher pressures, the gravimetric uptake in the case of PILS and SIFSIX-2-Cu is higher due to the larger pore dimensions of these systems.

The results obtained have also revealed that in the case of the CF_4_–SF_6_ fluid mixtures, under near-ambient pressure conditions, the carbon-based nanoporous materials exhibit a higher gravimetric fluid uptake and thermodynamic separation selectivity. On the other hand, the SIFSIX-2-Cu material exhibits a higher kinetic selectivity at both ambient and high pressures. Regarding the separation of the CF_4_–N_2_ mixtures, the carbon-based nanoporous materials exhibit a higher thermodynamic separation selectivity in comparison with the SIFSIX-2-Cu MOF but significantly lower in comparison with the values obtained in our previous studies for the SF_6_–N_2_ mixtures. However, as for the SF_6_–CF_4_ fluid mixture, the SIFSIX-2-Cu material also exhibits a higher kinetic selectivity at high pressures, in the range of 20 bar, in the case of the CF_4_–N_2_ mixture.

## Notes

The data that support the findings of this study are available from the corresponding author upon reasonable request.
